# Asymptomatic bronchial aspiration and prolonged retention of a capsule endoscope: a case report

**DOI:** 10.1186/1752-1947-5-341

**Published:** 2011-08-02

**Authors:** Alessandro Pezzoli, Nadia Fusetti, Alessandra Carella, Sergio Gullini

**Affiliations:** 1 Department of Gastroenterology and GI Endoscopy, University Hospital, Ferrara, Italy

## Abstract

**Introduction:**

Capsule endoscopy has, over the last few years, become a first-line test to visualize the mucosa of the small intestine. This technique is generally considered safe and does not cause discomfort for patients. However, although patients may have difficulty in swallowing the capsule, bronchial aspiration of a capsule endoscope is a very rare complication. We report the case of an 82-year-old man who experienced prolonged bronchial aspiration of a capsule endoscope without relevant symptoms, followed by a spontaneous return of the capsule to the gastrointestinal tract.

**Case presentation:**

An 82-year-old Caucasian man was referred to our unit from another local hospital to undergo capsule endoscopy. He swallowed the capsule without any apparent difficulties and did not show any overt symptoms. The following day, when we reviewed the capsule endoscopy images, we realized that the capsule was in the bronchial system and remained there for the duration of the study. An urgent X-ray of the chest confirmed the presence of the capsule in the left side of the bronchopulmonary tree. Two days later a repeat chest X-ray showed the capsule in the right bronchus. After two days the capsule was retrieved in the feces. Our patient remained asymptomatic during the entire admission period.

**Conclusions:**

Aspiration of a capsule endoscope is a rare complication; to the best of our knowledge this is the first reported case in which a capsule endoscope remained for six days in the bronchial system of a patient without causing airway compromise or pneumonitis and spontaneously returned to the gastrointestinal tract.

## Introduction

Capsule endoscopy is rapidly becoming a widespread tool used for small bowel exploration; the advantages of capsule endoscopy include the absence of discomfort for patients, good diagnostic yield and good safety profile. The main complication is capsule retention, reported in about 1% to 2% of cases [1,2]. Sometimes patients present difficulty in swallowing the capsule but bronchial aspiration of a capsule endoscope is a very rare complication, and only eight cases of bronchial aspiration have been reported in the literature [2-9]. We describe the case of a patient who experienced prolonged bronchial aspiration of a capsule endoscope without relevant symptoms, resolved with spontaneous return of the capsule to the gastrointestinal (GI) tract.

## Case presentation

An 82-year-old Caucasian man was referred to our unit from another local hospital in order to undergo capsule endoscopy. He presented with unexplained anemia, and previous upper and lower endoscopic examinations had failed to reveal any pathological findings. His medical history included arterial hypertension.

The patient swallowed the capsule in the presence of a physician without any evident swallowing difficulties. He then returned to the original hospital where the capsule endoscopy procedure was carried out. Our patient did not show any overt symptoms during the next day. The following day, the data recorder system was sent to our hospital to download the video.

**Figure 1:**
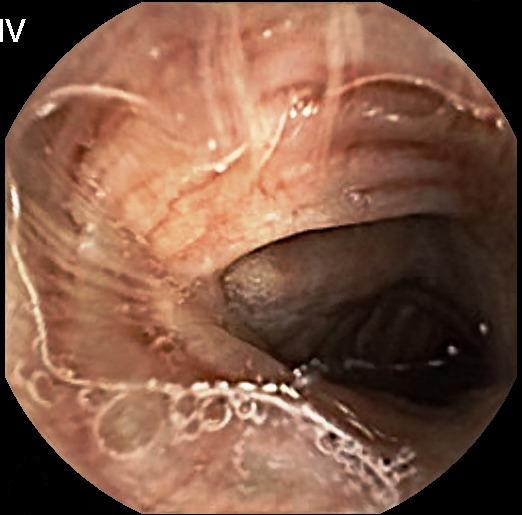
**Capsule endoscopy**. Capsule endoscopy view of the bronchial system.

**Figure 2:**
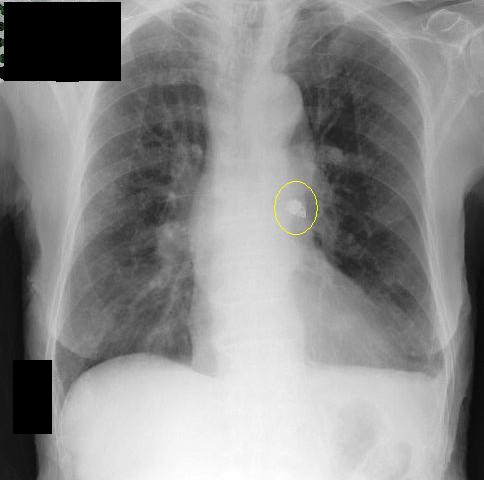
**X-ray of the chest**. X-ray of the chest confirming the presence of the capsule in the left side of the bronchopulmonary tree.

**Figure 3:**
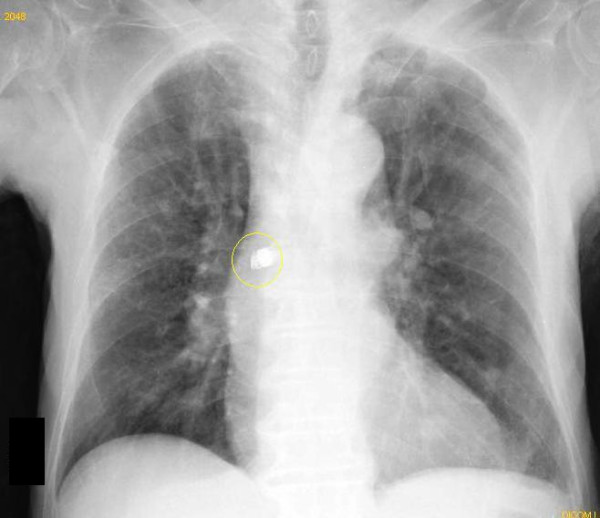
**Second X-ray of the chest**. Two days later, another X-ray of the chest was performed showing the capsule in the right bronchus.

When we reviewed the capsule endoscopy images we realized that the capsule was located in the bronchial system and had remained there for the entire duration of the study (Figure 1). We immediately contacted our colleagues from the other hospital who reported that our patient remained asymptomatic. An emergency chest X-ray confirmed the presence of the capsule in the left side of the bronchopulmonary tree (Figure 2). We proposed a bronchoscopy but our colleagues preferred a wait-and-see policy since our patient was asymptomatic apart from a minimal cough, with pulsed oxygen saturation of 96% in room air. Although the X-ray pictures were clear, another upper GI endoscopy was performed to check if the capsule was in the esophagus; the procedure results were negative. Two days later a repeat chest X-ray showed the capsule in the right bronchus (Figure 3). After two days the capsule was retrieved in the feces. Surprisingly, our patient remained asymptomatic during the entire admission period; he made an uneventful recovery and we decided not to repeat the capsule endoscopy procedure.

## Conclusions

Aspiration of capsule endoscope is a rare complication and only a few cases have been reported in literature [2-9]; the majority of the described cases occurred in older patients with no history of swallowing disorders; in two cases in two older patients (90 and 93 years old), no relevant symptoms were reported [5,7]. In general the routine use of the real-time video monitor to confirm the passage of the capsule in the esophagus is not recommended, but we think that in older patients this policy should be suggested. In our patient's case, he underwent an unnecessary upper GI endoscopy since our colleagues were not sure of the capsule position. Nowadays, new generation data recorders allow us to control, in real time, where the capsule is located. In one previous case a chest computed tomography (CT) scan was used to confirm the presence of the capsule in the bronchus system [3]. In some cases the capsule was removed by bronchoscopy [3,8], in the others spontaneous expulsion through coughing was observed, as in the case of our patient. However, this is the first case in which a capsule endoscope remained in the bronchial system for six days without causing airway compromise or pneumonitis. In our patient the capsule moved from one bronchus to another and finally returned to the stomach on coughing, without significant symptoms. Nonetheless, capsule aspiration could evolve into significant pulmonary complications; we suggest that, in case of non-spontaneous return of the capsule to the esophagus or in the presence of signs of respiratory distress, the capsule should be retrieved rapidly by bronchoscopy. The presence of difficulties in swallowing the capsule is not a predicting factor for aspiration, since in the majority of cases patients do not report such problems.

In summary, capsule endoscope aspiration is a rare but potentially life-threatening complication; nonetheless, it can occur without symptoms, mainly in geriatric patients, and can sometimes be spontaneously resolved.

## Consent

Written informed consent was obtained from the patient for publication of this case report and any accompanying images. A copy of the written consent is available for review by the Editor-in-Chief of this journal.

## Competing interests

The authors declare that they have no competing interests.

## Authors' contributions

AP carried out the capsule endoscopy procedure and drafted the manuscript. NF followed our patient during the admission period and helped to draft the manuscript. AC followed our patient during the admission period and helped to draft the manuscript. SG made substantial contributions to the manuscript and gave final approval of the version to be published. All authors read and approved the final manuscript.

## References

[B1] Li F Gurudu SR De Pretis G Sharma VK Shiff AD Heigh RI Post J Erickson P Leighton JA Retention of capsule endoscopy: a single-centre experience of 1000 capsule endoscopy procedures Gastrointest Endosc 2008 68 174 180 10.1016/j.gie.2008.02.037 18513723

[B2] Rondonotti E Herrerias JM Pennazio M Caunedo A De Franchis R Complications, limitations, and failures of capsule endoscopy: a review of 733 cases Gastrointest Endosc 2005 62 712 717 10.1016/j.gie.2005.05.002 16246685

[B3] Tabib S Fuller C Daniels J Lo SK Asymptomatic aspiration of a capsule endoscope Gastrointest Endosc 2004 60 845 847 10.1016/S0016-5107(04)02032-2 15557975

[B4] Sinn I Neef B Andus T Aspiration of a capsule endoscope Gastrointest Endosc 2004 59 926 927 10.1016/S0016-5107(04)00291-3 15173819

[B5] Buchkremer F Herrmann T Stremmel W Mild respiratory distress after capsule endoscopy Gut 2004 53 472 10.1136/gut.2003.033845 14960546 PMC1773982

[B6] Shiff A Leighton JA Heigh RI Pulmonary aspiration of a capsule endoscope Am J Gastroenterol 2007 102 215 216 17278275

[B7] Nathan SR Biernat L Aspiration-an important complication of small-bowel video capsule endoscopy Endoscopy 2007 39 E343 10.1055/s-2007-995327 18273788

[B8] Guy T Jouneau S D'Halluin PN Lena H Asymptomatic bronchial aspiration of a video capsule Interact Cardiovasc Thorac Surg 2009 8 568 570 10.1510/icvts.2008.190058 19246497

[B9] Leeds JS Chew TS Sidhu R Elliot CA Sanders DS McAlindon ME Asymptomatic bronchial aspiration and retention of a capsule endoscope Gastrointest Endosc 2009 69 561 10.1016/j.gie.2008.09.044 19058800

